# A call for a paradigm shift to community-embedded, home-based primary preventive oral health care in Africa

**DOI:** 10.3389/froh.2025.1751738

**Published:** 2026-01-12

**Authors:** Moréniké Oluwátóyìn Foláyan, Ahmed Bhayat, Maha El Tantawi

**Affiliations:** 1 AFRONE Network, Faculty of Dentistry, Alexandria University, Alexandria, Egypt; 2 Early Childhood Caries Advocacy Group, University of Manitoba, Winnipeg, MB, Canada; 3 Oral Health Initiative, Nigerian Institute of Medical Research, Lagos, Nigeria; 4 Department of Child Dental Health, Obafemi Awolowo University, Ile-Ife, Nigeria; 5 Department of Community Dentistry, University of Pretoria, Pretoria, South Africa; 6 Department of Pediatric Dentistry and Dental Public Health, Faculty of Dentistry, Alexandria University, Alexandria, Egypt; 7 Faculty of Dental Medicine, Alamein International University, New Alamein, Matrouh, Egypt

**Keywords:** community engagement, health disparities, home-based intervention, multisectoral governance, preventive dentistry, sustainable health practices, telehealth, universal health coverage

## Introduction

Oral diseases constitute a global public health crisis, affecting nearly 3.5 billion people worldwide (1). They cause pain, disability, and economic loss, with their highest burden falling on vulnerable and marginalized populations (1, 2). This crisis is exacerbated by a widespread systemic failure: c (3). This exclusion frames oral health as a discretionary rather than an essential service, rendering cost a primary barrier to access (4). Africa exemplifies the severe consequences of this global neglect. The continent is projected to experience the largest relative increase in oral diseases by 2030, driven by urbanization, dietary changes, and the persistent low prioritization of oral health (3, 5, 6). The prevailing clinic-centric, curative model is not only misaligned with regional demographics, resource constraints, and cultural contexts (7) but also replicates a global paradigm that uses high cost as a rationale for exclusion rather than as an impetus for innovating equitable, preventive solutions. Therefore, the challenge in Africa is a pressing demonstration of the need for a paradigm shift in how oral health is valued and delivered.

The persistent inadequacy of oral health care in Africa is a deeply entrenched structural problem, rooted in systemic and self-perpetuating deficiencies that are more than a simple shortfall of resources or logistics. This structural failing is evident in the severe maldistribution and shortage of the oral health workforce, where the low dentist-to-population ratio, frequently reaching 1:100,000, stands in stark contrast to ratios around 1:2,000 in high-income countries (2, 6). This scarcity is exacerbated by a maldistribution of care providers, with most dental facilities concentrated in urban centers (8). Consequently, rural populations, which constitute the majority in many African countries (9), face geographic barriers to accessing care (10). Compounding this is the policy and financing neglect, as oral health remains excluded from most UHC schemes and primary care policies (11), resulting in catastrophic out-of-pocket expenditures for many households (12). In the absence of political will and dedicated preventive funding, the oral health burden is normalized for marginalized populations due to a lack of (11–13).

In the absence of accessible and affordable professional care, populations often resort to traditional or complementary remedies (14). While these remedies are often effective for basic hygiene, they lack an evidence base for managing acute conditions, potentially leading to delayed presentation and health complications (15). Yet, the prevailing clinic-centric model, derived from Western biomedical frameworks, creates a systemic cultural and institutional disconnect by overlooking and not strengthening indigenous health practices and community-based oral health care traditions, thereby widening the access gap (15, 16). The cumulative effect is a situation where a preventable condition like dental caries can progress to a life-threatening infection and chronic oral pain (17).

This disconnect is exacerbated by infrastructural and technological inequities, where limited preventive public health infrastructure and uneven digital access reinforce disparities rather than resolve them (18). The cycle is perpetuated by dental education curricula that remain focused on curative, clinical care, offering minimal training in community-based prevention, cultural competence, or the integration of traditional knowledge systems (16). Collectively, these intertwined factors create a system in which oral diseases are treated late (19–21), and late access to treatment is systematically sustained by the very structures designed to combat them.

While it is known that prevention remains the most viable option to address oral disease (1) and a strategic necessity to achieve health equity, reduce healthcare costs, and improve overall quality of life (22). The critical question is how to implement it effectively within the African socio-economic and cultural landscape. Mitigating the oral health crisis in Africa requires fundamental structural reorientation toward community-embedded, preventive, and culturally intelligent models of care. The aim of the study is to advocate for a shift away from the current prevailing model of oral healthcare in Africa, and to propose a new, community-embedded, home-based primary preventive model.

### The need for a new prevention paradigm for households in Africa

The conventional oral healthcare model, which relies on encouraging clinic attendance for preventive check-ups and education, is rooted in a Western biomedical framework and fails to account for the geographic, financial, and cultural barriers that limit clinic access, thereby exacerbating health inequities. It is ill-suited to the infrastructure, economic realities, and cultural practices of many low-resource settings in Africa as it inherently places the onus of cost, time, and travel on the individual, thereby disproportionately disadvantaging rural and low-income communities. This raises a pivotal question: in settings where transportation costs are significant considerations, and where cultural beliefs often prioritize home-based and community-advocated remedies (15, 16), is a clinic-based model for prevention justifiable?

For Africa, where a more equitable, efficient, and culturally intelligent model of delivering oral health care is needed, alternative(s) must be explored. A viable path forward is a reorientation towards home-based and community-driven primary prevention. This is a call to re-engineer the ecosystem, positioning the household as the primary locus of prevention, supported by a strengthened public health infrastructure that actively incorporates indigenous knowledge and hands the responsibility of prevention to empowered communities. This model aligns with the WHO's building blocks approach by focusing on community ownership and integrating oral health into broader health initiatives (23).

Advocating for a home-based, community-embedded model of oral health prevention does not diminish the fundamental responsibility of governments and health systems to ensure population health. Rather, this paradigm shift represents a strategic reorientation toward primary prevention, anchored by robust public health infrastructure, targeted professional development, and equitable distribution of tools and knowledge. The model's success wholly depends on this systemic support; without it, responsibility is devolved onto individuals and communities, exacerbating the very inequities it seeks to address. The proposed model, therefore, envisions a synergistic partnership, positioning the household and community as the frontline of daily prevention—actively fortified by the state through funded preventive programs, subsidized access to essentials like fluoride toothpaste, and culturally sensitive training curricula (24). Ultimately, the goal is to avoid making people responsible for outcomes beyond their control due to inadequate support. Instead, the model empowers them within a framework of shared responsibility, where public health infrastructure enables effective self-care and community action, thereby transforming the locus of care without abandoning the population.

### The pillars of a home-based community-embedded prevention model

This proposed model offers several distinct advantages, with an emphasis on cultural leverage. First, a preventive approach is economically prudent. Evidence indicates that population-based preventive programs yield a high return on investment: for every USD invested in community-based oral disease prevention, up to USD 50 in treatment costs can be saved (25). Empowering individuals, households, and communities with the knowledge and tools for effective self-care, including the use of affordable fluoride toothpaste, sugar consumption limitation, and other dietary strategies, can prevent a significant proportion of oral diseases, thereby alleviating financial pressure on both households and the overstretched public health systems.

Second, a key, yet underutilized, strategy is the respectful engagement with and leverage of culturally resonant health promotion, often embodied by community-based actors like traditional healers. As demonstrated in Yorùbá culture, songs, proverbs, and folklore are powerful tools for embedding health messages (15, 16). Public health campaigns can leverage these existing oral traditions to disseminate messages about oral hygiene, reframing modern practices like fluoride toothpaste use within the context of cultural values such as spiritual balance, personal dignity (*iyi*), and social respectability (16).

Although the integration of culturally resonant health promotion is often promoted through the integration of traditional healers into formal health systems (26), this integration approach remains unresolved, facing challenges related to training standardization, regulatory frameworks, and scope of practice (27, 28). Our proposal does not suggest that traditional healers can or should replace absent dentists or solve systemic human resource shortages in isolation. Rather, it advocates for a complementary and collaborative model. Community Health Workers (CHWs) and respected community figures, including some traditional healers who share a language and worldview with the community, can be trained as oral health promoters. Their role would be to champion the complementary use of evidence-based prevention alongside trusted cultural practices, such as the use of chewing sticks with known antimicrobial properties (29, 30). This can bridge the trust gap and transform cultural barriers into facilitators.

The proposed model is not an alternative to building a robust health workforce but a necessary, parallel investment to extend its reach and impact sustainably. It seeks to “task-share” health promotion and simple prevention, not “task-shift” the full burden of clinical care. This strategy needs to use a dual approach: (1) Strengthening the economic, human, and physician resources of the formal oral health system remains a non-negotiable priority for managing disease and handling referrals. (2) Simultaneously, empowering community-based networks to deliver primary prevention. This decolonizes the approach by valuing indigenous knowledge systems while anchoring them within a strengthened public health infrastructure that can provide oversight, training, and a clear referral pathway.

Third, the environmental impact of health care delivery is an emerging concern. Clinic-based models necessitate frequent patient travel, consuming fuel, and generating carbon emissions (31). Furthermore, complex restorative treatments for advanced disease consume more materials and energy, contributing to medical waste (32). A home-based prevention model inherently reduces the carbon footprint of oral health care by minimizing unnecessary travel, reducing disease incidence, and diminishing the demand for resource-intensive curative procedures (33).

Fourth, digital health technologies present a transformative opportunity to overcome geographic barriers. The proliferation of mobile phone networks enables the use of tele-dentistry, allowing CHWs to perform basic screenings and receive remote guidance from dental professionals (34). Smartphone penetration in Africa is growing rapidly, increasing from 64% in Sub-Saharan Africa in 2024 to 75% in 2025 (35). Mobile health (mHealth) applications can deliver standardized oral hygiene instruction, including culturally tailored animations using local music and proverbs, dietary advice, and preventive reminders in local languages. This task-shifting and tele-guidance approach, validated in programs for oral health (36), can expand the reach of oral healthcare systems without requiring more dentists (37).

Table 1 provides a summary of the argument presented in the paper by systematically contrasting the limitations of a clinic-centric model with the proposed household-focused, community-supported alternative preventive model. The paradigm shift is a move from a clinic-centric model to one that relocates the primary locus of preventive oral healthcare by empowering and enhancing self-care at the individual household level, which is reinforced by a network of support within the community.

**Table 1:** Contrasting models of oral healthcare for the African context.

Feature	Current prevailing model (clinic-centric, curative)	Proposed paradigm shift (community-supported, home-based prevention)
Primary locus	Dental clinic/Hospital	Household and Community
Core focus	Treatment of established disease	Primary prevention before disease onset
Key actors	Dentists, Dental Therapists, Oral Healthcare Professionals, systems support	Individuals, Community Health Workers, Traditional healers, Teachers, Caregivers, supported by Oral Healthcare Professionals and Government-funded programs, professional training, resource allocation, and policy frameworks
Cultural approach	Western biomedical framework, often dismissive of indigenous practices	Culturally intelligent; leverages and integrates indigenous knowledge, songs, and proverbs
Technology use	Limited to clinical equipment	Tele-dentistry, mHealth apps for screening, remote guidance, education, and reminders
Economic impact	High cost for patients; high cost for healthcare systems	Cost-saving; high return on investment through prevention
Equity and access	Exacerbates inequities; favors urban, affluent populations	Improves equity; reaches rural and low-income populations where they live
Environmental impact	Higher carbon footprint due to patient travel and resource-intensive procedures	Lower carbon footprint by minimizing travel and reducing the need for complex treatment
Integration	Often siloed from general health and primary care	Strategically integrated into primary care and broader public health initiatives
Defining goal	Manage disease episodes	Empower individuals, households, and communities for sustained health and well-being

This is achieved through several key changes outlined in the table, including the shift from the professional dental clinic to the household, establishing it as the frontline for daily prevention, while the community becomes the supportive ecosystem. Key actors are expanded beyond oral healthcare professionals to include individuals, caregivers, CHWs, teachers, and traditional healers. This creates a multi-layered support system that guides, educates, and reinforces positive self-care practices within everyday life. The approach becomes culturally intelligent, leveraging indigenous knowledge, oral traditions, and technology to make evidence-based self-care practices more resonant, sustainable, and responsive to local resources. The proposed model transforms prevention from a periodic clinical event focused on managing disease by an external authority (the dentist) into a continuous, community-embedded practice for sustained health and well-being managed by empowered individuals.

### Systemic challenges and implementation considerations

Implementing this paradigm shift faces significant systemic challenges that demand strategic and context-sensitive solutions to prevent the inadvertent perpetuation of health inequities. First is the need for sustained political will to reorient national health budgets from a curative to a preventive focus, including the integration of oral health into UHC schemes and primary care policies (11). Innovative financing, such as earmarked taxes on sugar-sweetened beverages shown to be effective in Africa (38), alignment of donor investments with frameworks like the African Union's Agenda 2063 through domestication into national development plans, coordinated partnerships, and leveraging various financing mechanisms (39), and the reallocation of funds from high-cost tertiary procedures to community-based prevention can yield a high return on investment for this program.

Second, addressing workforce gaps requires scaling training for CHWs and traditional healers without compromising quality. This necessitates a standardized, accredited curriculum focused on core preventive competencies (identifying early signs of caries and gingivitis, delivering motivational interviewing for fluoride toothpaste use and sugar reduction, using a validated mobile app for risk assessment and capturing images for tele-consultation, and applying a simple referral algorithm based on symptoms to designated primary care dental units), supported by ongoing supervision and tele-mentorship from dental professionals (37), alongside continuous professional development and clear scope-of-practice guidelines to ensure safety and clarity (27, 28). The digital divide presents another barrier; although smartphone penetration is rising (35), uneven access and literacy risk widening disparities (18). A hybrid approach combining mHealth apps for connected users (36) with low-tech solutions like SMS reminders, interactive voice response, and community radio in local languages, shown to be effective (40), is essential for inclusive reach.

Furthermore, tele-dentistry and community screening must be formally linked to clinical care through structured referral protocols. Clear guidelines, supported by mobile diagnostic tools for CHWs (34), should define urgent red-flag conditions, establish feedback loops for follow-up, and integrate seamlessly with existing primary care networks to avoid siloed systems. Finally, cultural and logistical sensitivities must be navigated with care. Potential resistance from professional associations can be mitigated through the co-creation of guidelines and evidence-sharing. Respect for the intellectual property of traditional knowledge, credited inclusion of cultural custodians in program design (16), and transparent, long-term community engagement are all vital to building trust and reinforcing the complementary, not replacement, role of cultural practices within an evidence-based prevention framework. The goal is to establish every household as its own first line of defense, supported by the consistent practice of evidence-based prevention, such as twice-daily use of fluoride toothpaste and reduction in sugar consumption, which remains one of the most effective public health interventions for dental caries (41, 42), while respecting and incorporating culturally validated self-care practices.

To realize this vision, a phased, adaptive implementation strategy is essential to pilot, refine, and scale the model effectively as outlined in Table 2. By deeply embedding prevention into community and primary care structures, a resilient oral health ecosystem capable of enduring with reduced reliance on external funding can be fostered. Successful implementation requires multisectoral governance, actively involving Ministries of Health, Education, Local Government, and Finance. National Oral Health Prevention Guidelines must be co-developed with communities and strategically aligned with overarching frameworks such as the WHO's Framework for Integrated People-Centred Health Services (43) and the African Union's Health Strategy 2016–2023 (44). A robust monitoring and evaluation framework is needed to track progress through key indicators like the reduction in early childhood caries incidence, coverage metrics such as the proportion of households with access to affordable fluoride toothpaste, workforce data on the number of trained CHWs and traditional healers per district, and perceived quality measures of patient satisfaction and cultural relevance. Further strengthening resilience would require promoting the local production of affordable fluoride toothpaste to ensure a reliable supply, create economic opportunities, and reduce import dependence (45, 46).

**Table 2:** Phased implementation roadmap for a community-embedded oral health prevention model.

Phase	Focus	Key activities	Outcomes
Phase 1: Pilot and Learn (Years 0–2)	Select communities with existing CHWs and traditional healers’ networks.	Train CHWs and traditional healers; deploy hybrid mHealth tools; pilot tele-dentistry referrals.	Proof of concept, community feedback, and refined training modules.
Phase 2: Integrate and Expand (Years 2–5)	Integrate oral health into primary care platforms (e.g., maternal and child health visits).	Develop referral pathways, engage local government, and scale training via health training institutions.	Improved access metrics, policy dialogue initiated, and early impact on caries incidence.
Phase 3: Scale and Sustain (Years 5–10)	National scale-up with policy integration.	Adopt national oral health prevention guidelines, reform dental curricula, and establish a monitoring and evaluation system.	Sustainable funding, reduced oral disease burden, and an empowered community health workforce.

## Conclusion

It is time to move beyond attempting to retrofit a Western, clinic-centric model onto the complex realities of Africa. The future of oral health in Africa lies in decentralizing prevention, democratizing care, leveraging technology, integrating with primary care, and, most importantly, empowering communities by drawing upon their immense cultural and human capital. This model reimagines health systems as enablers of prevention. It calls for governments, professionals, and communities to co-create a cost-effective, sustainable, and scalable oral health ecosystem where prevention is accessible, culturally resonant, and systematically supported as an ethical and practical imperative to mitigate the growing epidemic of oral disease on the continent. Ultimately, the sustainability of this paradigm shift hinges on parallel reforms in dental education, producing graduates who are as proficient in cultural humility and community partnership as they are in clinical science.
